# 吞咽困难为主诉的原发性支气管肺癌1例报道

**DOI:** 10.3779/j.issn.1009-3419.2011.06.15

**Published:** 2011-06-20

**Authors:** 核兰 王, 继陶 周, 黎 魏, 梅 李, 锋 罗

**Affiliations:** 1 610041 成都，四川大学华西医院肿瘤中心，生物治疗国家重点实验室，头颈乳腺肿瘤科和肿瘤内科 Department of Head & Neck and Mammary Oncology and Department of Medical Oncology, Cancer Center and State Key Laboratory of Biotherapy, West China Hospital, Chengdu 610041, China; 2 610041 成都，四川省林业中心医院 Forestry Central Hospital of Sichuan, Chengdu 610041, China

原发性支气管肺癌首发常见症状是咳嗽、咳痰、咯血等呼吸道表现。吞咽困难是食管癌的典型临床症状，极少数原发性支气管肺癌患者由于肿瘤进展在病程后期可以出现此症状。本文报道1例特殊的肺癌病例，患者以吞咽困难为首发症状就医，胃镜检查发现食管粘膜隆起、表面溃疡，病理活检提示鳞状细胞癌，以食管癌收入院，行进一步检查后确诊为原发性支气管肺癌。

## 1 临床资料

患者男性，71岁，因出现吞咽固体食物梗阻1月就医，自述梗阻部位位于剑突下，门诊行胃镜示距门齿35 cm食管中段处粘膜隆起，表面粘膜破溃（图 1A），病理活检示鳞状细胞癌，考虑为食管癌收入胸外科。入院后进一步追问病史，患者除前述症状外，无其它消化道症状，亦无咳嗽、咳痰、咯血、胸痛、胸闷等呼吸道症状，发病后体重无明显变化。平素健康状况良好，有吸烟史50年，饮酒史30年。查体：生命体征平稳，一般情况可，全身浅表淋巴结未触及肿大，肺部触诊触觉语颤正常，叩诊过清音，听诊双肺呼吸音粗，双下肺闻及细湿啰音，心脏、腹部查体未见异常。食道钡餐检查示中纵隔偏右见团块影，约8.7 cm×4.5 cm，伴右肺门影增浓，食管明显受压左移，局部管壁欠规则，可疑粘膜破坏，性质待定。胸部CT发现食管中下段管壁增厚，与周围肿大淋巴结分界不清；右肺门、纵隔多发淋巴结肿大并融合成块；右肺下叶背段感染灶（图 2）。纤维支气管镜检查示右肺下叶背段支气管开口菜花样新生物阻塞（图 1B），病理活检示右肺下叶背段查见鳞状细胞癌。肿瘤标志物检查示CEA为5.60 mmol/L，NSE为25.43 mmol/L，CYFRA21-1为3.12 mmol/L。腹部CT及头部MRI未见明显异常。为进一步明确肿瘤的组织学类型及来源行免疫组织化学染色，食管中段活检肿瘤组织细胞示CK7（-），CK20（-），TTF-1（-）；支气管右下叶背段活检肿瘤组织示CK7小灶区（+），CK20（-），TTF-1灶性少数细胞（+），免疫组织化学染色提示肿瘤来源于肺可能性大（图 3）。诊断为右肺鳞癌伴右肺门、纵隔淋巴结、食管转移T4N3M0 Ⅲb期。患者无手术指征遂转入肿瘤科，给予TP方案（紫杉醇+顺铂）化疗4周期后评价疗效为进展。因放疗风险患者拒绝行放射治疗，治疗方案改为培美曲塞，化疗2周期无效后改为口服厄洛替尼（特罗凯）治疗，目前已随访6个月患者仍生存。

**1:**
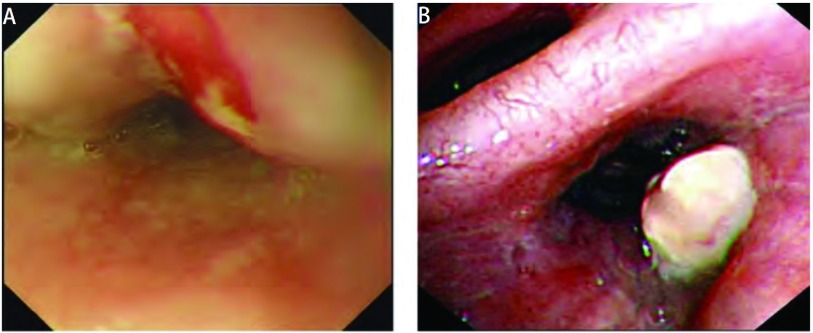
胃镜及纤维支气管镜检查。A：胃镜查见食管中段处粘膜隆起，表面粘膜破溃；B：纤维支气管镜检查见右下叶背段支气管开口菜花样新生物。 Gastroscope and Fiberoptic bronchoscopy. A: Gastroscope: the mucosal bulge with ulceration in the middle esophagus; B: Fiberoptic bronchoscopy: neoplasm like cauliflower in the lower right dorsal segment of bronchus.

**2:**
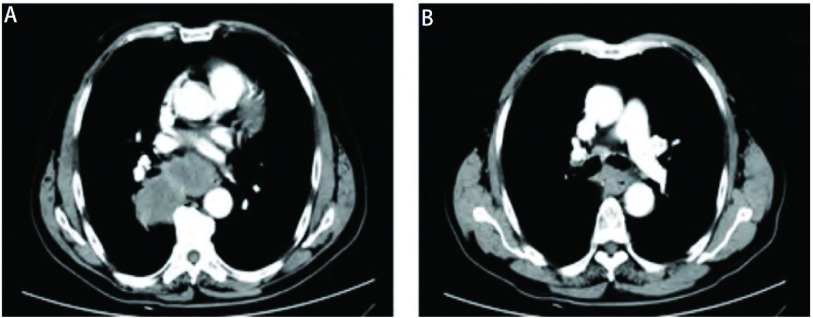
胸部CT。A：右肺门、纵隔淋巴结增大融合；B：食管中下段管壁增厚。 Computed tomography of the chest. A: The fused enlargement of lymph nodes in right hilar and mediastinum; B: The thickened wall of lower esophagus.

**3:**
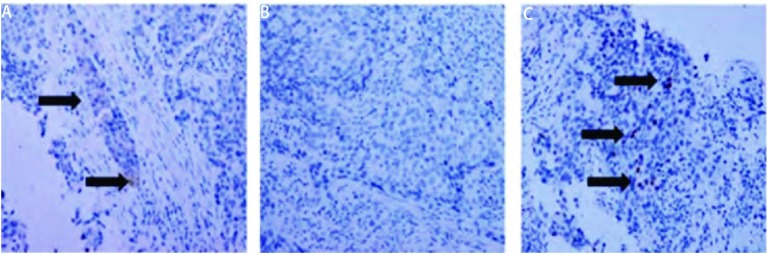
支气管右下叶背段活检组织免疫组化染色（SABC, ×200）。A：CK7小灶区（+）; B：CK20（-）; C：TTF-1灶性（+）。 Immunohistochemistry of the tissue section of the lower right dorsal bronchus(SABC, ×200). A: CK7 (focal positive); B: CK20(-); C: TTF-1 (focal positive).

## 2 讨论

原发性支气管肺癌是目前全世界最常见的恶性肿瘤，流行病学研究^[1]^显示其发病率及死亡率均居全球恶性肿瘤首位。近年来我国的肺癌发病率仍呈上升趋势。该病临床表现复杂，早期缺乏典型症状，容易延误诊断，大多数患者确诊时已失去手术机会。Spiro等^[2]^认为详细研究肺癌患者首发症状对早期诊断有重要意义，肺癌患者发病之初多出现呼吸道相关症状，最常见为咳嗽（8%-75%）、气促（3%-60%）、胸痛（20%-49%）及咯血（6%-35%），消瘦、乏力等非特异性症状也较为常见，少部分患者出现转移病灶引起的首发症状如骨痛等，仅有 < 2.2%的肺癌患者会出现吞咽困难的症状，且多于疾病后期出现，是病情进展的表现。另有学者^[3-6]^进行大量病例分析后发现，肺癌患者病程中出现吞咽困难的原因最常见为纵隔肿瘤外压食管，其次是颈部淋巴结转移压迫上段食道，较少见的是前纵隔放射治疗后引起。此外有学者^[6, 7]^补充了另外5种较为罕见的原因：脑转移、消化道转移、系统性病变如皮肌炎累及食道、口咽部及食道真菌感染和二重癌。

本例患者发病时吞咽困难为唯一症状，首诊时胃镜活检示鳞状细胞癌，以食管癌收住院，随后行纤维支气管镜检查发现新生物，活检为鳞状细胞癌，胸部CT提示纵隔肿块，考虑肺癌可能性大。但鉴别诊断仍需病理免疫组化分析。TTF-1是一种核转录因子，TTF-1蛋白在甲状腺滤泡上皮细胞、肺的细支气管上皮和Ⅱ型肺泡上皮中表达，特异性表达于肺癌及甲状腺癌组织^[8]^。CK7是一种碱性角蛋白，主要在肺、乳腺、子宫内膜等正常组织及肿瘤组织中表达，CK20是一种酸性角蛋白，表达于胃肠道、胆道、尿道的正常组织及肿瘤组织^[9]^。文献^[10-12]^报道利用TTF-1、CK7和CK20三种蛋白的不同表达情况可较为准确的鉴别肺原发性癌及转移性癌，如CK7（+）伴TTF-1（+）高度提示肺原发性癌，CK7（-）、CK20（+）伴TTF-1（-）提示为肺转移性癌，且多来源于消化道肿瘤转移，而CK7（+）、TTF-1（-）时考虑肺转移性乳腺癌可能性大。本例患者所取两处组织的免疫组化检测结果有差异，食管中段活检组织肿瘤细胞CK7（-）、CK20（-）、TTF-1（-）；支气管右下叶背段活检组织肿瘤细胞CK7小灶区（+）、CK20（-）、TTF-1灶性少数细胞（+），考虑为肿瘤细胞本身的异质性及免疫组化实验组织处理条件不同造成，肺转移性癌的可能性较小，结合临床其它证据最后诊断为原发性支气管肺癌。

本例患者没有肺癌典型临床表现，以吞咽困难就诊，最终通过CT、纤维支气管镜及病理活检免疫组化分析后确诊为原发性支气管肺癌。原发性支气管肺癌缺乏特异性症状，尤其是纵隔型肺癌常有吞咽困难的症状，所以在肿瘤诊断过程中需充分掌握影像学资料，另外利用免疫组化鉴别肿瘤组织来源在诊断中也有极其重要的作用。
